# PTSD and challenges among older Chinese in Shenzhen during COVID-19 pandemic: Trust in authority and medical professionals as moderators

**DOI:** 10.1017/S1463423624000641

**Published:** 2025-01-09

**Authors:** Jiahui Jin, Daniel W.L. Lai, Vincent W.P. Lee, Elsie Yan, Alison X.T. Ou, Julia Juan Wang

**Affiliations:** 1 Wee Kim Wee School of Communication and Information, Nanyang Technological University, Singapore, Singapore; 2 Faculty of Arts and Social Sciences, Hong Kong Baptist University, Hong Kong, China; 3 Shenzhen Elderly Healthcare College, Shenzhen Polytechnic University, Shenzhen, China; 4 Department of Applied Social Sciences, The Hong Kong Polytechnic University, Hong Kong, China

**Keywords:** COVID-19, older adults, aging, PTSD, trust

## Abstract

**Aim:**

This research aimed to comprehensively explore the impact of diverse challenges encountered by older adults on the development of post-traumatic stress disorder (PTSD). It delved into how these effects vary depending on individuals’ levels of trust in authority and medical professionals, providing a nuanced understanding of the interplay between external challenges, personal trust, and mental health outcomes in the older population.

**Background:**

The COVID-19 pandemic has imposed significant hardships, particularly on the ageing population, with potential psychological repercussions such as PTSD. Notably, there is a dearth of research exploring this association within the context of Chinese older adults, a group that may experience unique impacts due to cultural differences in the face of global crises.

**Methods:**

Data were collected from a representative sample of 1,211 participants aged 60 years and above in Shenzhen. Logistic and hierarchical linear regression methods were utilized to investigate the relationship between the challenges posed by COVID-19, public trust, and the manifestation of PTSD symptoms.

**Findings:**

Higher levels of challenges related to ‘supplies, services access and safety’, ‘abuse and conflicts’, and ‘anger and fear’ were associated with PTSD. Furthermore, a lower level of challenges related to ‘disease management and information’ was associated with PTSD. Trust in authority or medical professionals was the moderator between the challenges brought about by COVID-19 and PTSD, which helped to lower the impact of challenges. Despite the challenges brought by COVID-19 to people, nurturing a stronger sense of trust in authority and medical professionals would ease older adults’ psychological stress and concerns.

## Introduction

According to the World Health Organization (WHO) data, as of May 2022, there were more than 500 million confirmed cases of Coronavirus Disease 2019 (COVID-19), including more than 6 million deaths (WHO, 2022). COVID-19 continues to pose a health threat, has dramatically disrupted the day-to-day lives of many individuals, and has negatively affected mental health (Xiong *et al*., 2020). Moreno *et al*. (2020) suggested that the unpredictability and uncertainty of COVID-19 and containment strategies placed a mental burden on the public. Concerns about being infected were reportedly the strongest predictor of anxiety, depression, and other negative emotions (Chen *et al*., 2021; Han *et al*., 2021; Li *et al*., 2020). Being female, having chronic diseases, and belonging to younger age groups were factors reportedly more likely associated with psychological symptoms (Ahmed *et al*., 2020). Numerous studies, scientific discourses, and news broadcasts have reported older adults’ vulnerability to COVID-19 and resultant deaths, highlighting the fact that older adults are at a higher risk of experiencing amplified ageism and under greater pressure (Collaborative *et al*., 2020; Landry *et al*., 2020; Werner *et al*., 2022).

Recent findings have revealed that COVID-19 has affected older adults’ life satisfaction, primarily through its negative influence on personal health, personal relationships, and standard of living, which are the three most central domains influencing satisfactory lifestyles among older adults (Chen and Olsen, 2022). The psychological impact of COVID-19, such as loneliness, was widely regarded as a consequence of restrictive protective measures and isolation (Jaspal and Breakwell, 2022; Yan *et al*., 2022). As highly suggested measures, social distancing policies create immense challenges for older adults, who are constrained from visits by family members and others, restricting their social participation (Sepúlveda-Loyola *et al*., 2020). Webb and Chen (2022) found that rates of anxiety and depression among older adults have increased because of social isolation during the pandemic, negatively impacting their quality of life, functioning, and general health. Additionally, preventive measures, such as restricting physical activity and changing dietary habits, have negatively impacted the daily lives of older adults (Kinoshita *et al*., 2022). While several countries and regions have adopted the policy of co-existing with COVID-19, China has adopted the Find, Test, Trace, Isolate, and Support; or the so-called Zero-COVID (FTTIS) policy with stricter containment strategies, such as extended quarantine duration and repeated testing (Chung *et al*., 2021). Particularly, the adoption of a ‘lockdown’ in the areas with cases has greatly disrupted the daily lives of residents. Additionally, ‘infodemics’ caused by overwhelming information about the spread of the virus from social media have also created severe psychological problems (Srifuengfung *et al*., 2021).

Post-traumatic stress disorder (PTSD) is regarded as ‘the second tsunami of the SARS-CoV-2 pandemic (Dutheil *et al*., 2021).’ Suicidal ideation, as the worst consequence of PTSD, has attracted the attention of researchers studying the impact of COVID-19 (Sher, 2020). While caution must be exercised to recognize the causes of suicide, there has been a significant uptick in the suicide rates during the pandemic, and a higher average rate of access mortality was found among individuals aged over 70 years (Watanabe and Tanaka, 2022). Some scholars believed that serious psychological disorders such as PTSD are more prevalent among older adults during the pandemic (Vrach and Tomar, 2020). In most studies examining PTSD and COVID-19, the target populations were mainly healthcare workers and infected individuals (Dubey *et al*., 2020). However, loneliness, which remains the key psychological trauma experienced by older adults, increases greatly among the aging population, primarily because of psychotic symptoms, relationship problems, and problems with daytime activities during COVID-19 (Greig *et al*., 2022). Furthermore, there is limited research on the relationship between the impact of COVID-19 on daily life and PTSD. More research is needed to facilitate the development of appropriate interventions aimed at reducing the long-term psychosocial effects of the pandemic.

Bennett (2020) stated that the success of public health responses to the COVID-19 pandemic is sensitive to public trust in experts. In addition to the acceptance of preventive measures, the psychological impact of the pandemic was found to be significantly linked to trust in experts. Trust in experts (e.g., government, media, health care institutions), as a protective factor against psychological problems, was found to have the ability to control the negative impact of the pandemic (Mohammadi *et al*., 2020; van Tilburg *et al*., 2021). Therefore, Georgieva *et al*. (2021) recommended building public trust to minimize the psychological problems caused by COVID-19. Nevertheless, only a few studies in the current literature have assessed public trust using Mainland Chinese samples. Unlike Western countries, the Chinese public maintained a relatively high level of political trust resulting from institutional performance and government-controlled politicization (Wang, 2005; Yang and Tang, 2010). Yang and Tang (2010) suggested that high public trust helps governments or institutions operate effectively, suggesting that the public prefers to follow the advice from the authorities under non-coercive conditions, which might increase public compliance with preventive measures and reduce disappointment towards the government. As the first country to suffer from COVID-19, China maintained strict preventive measures; subsequently, increased psychological stress was reported in the population (Li *et al*., 2021). High levels of public trust and stringent measures lead to higher compliance and a greater burden (Lin *et al*., 2022; Rivera-Torres *et al*., 2019; Yang and Tang, 2010). In a study in Iran, where people had lower trust in the government and national media, trust in authority significantly reduced psychological problems during the pandemic (Mohammadi *et al*., 2020). However, the applicability of this finding to China remains unknown because of the differences in the socio-political and regional cultural contexts in which public trust emerges. Thus, it is worth examining the role of public trust towards the government and authorities because of their role in initiating disease prevention policies and restrictions affecting everyone’s daily routines in different regions. Additional research findings will also serve to verify or validate the findings identified in previous studies in different socio-political contexts.

Considering the vulnerability of the aging population in this global pandemic, the present study examined the association between PTSD and challenges in various aspects of life brought about by COVID-19 and identified the role of trust in this relationship in China. In this study, we tested two hypotheses.

H1: Facing more challenges brought about by COVID-19 is associated with higher levels of PTSD.

H2: Trust in authority/medical professionals acts as a moderator, mitigating the effects of challenges on the level of PTSD.

## Methods

### Research design

A large-scale quantitative survey was conducted in Shenzhen between 23 July 2020 and 7 August 2020. To maintain statistical representative, we calculated our target sample size (1,066) using a 95% confidence interval and a 3% margin of error, applying the relevant formula. To account for potential unforeseen data omissions, we included 1,211 respondents in our study.

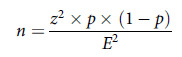




The inclusion criteria were Chinese people aged 60 years or above living in Shenzhen, able to communicate in Mandarin, and without cognitive impairment. A total of 60 residents’ committees were randomly identified in 10 administrative districts of Shenzhen, and the number of residents’ committees in each administrative district was determined according to the distribution of the resident population in Shenzhen. Residents’ committees in each administrative district were randomly selected, and trained interviewers were assigned to conduct random stops or door-to-door interviews in selected communities. Each resident committee interviewed 20 participants. Participants’ consent was obtained before the interview, and each survey’s questioning process was recorded. The one-on-one interviews ensured that the missing values were kept to a minimum during data collection. This study was conducted in collaboration with a local research institution and was approved by the university’s research ethics committee.

### Measurements

The questionnaire included demographic questions (such as gender, age, and education), physical health conditions, challenges faced during COVID-19, trust in authority and medical professionals, and PTSD. Physical health was assessed using a five-point Likert scale ranging from 1 (very bad) to 5 (very good). Trust in authority and trust in medical professionals were evaluated using two items rated on an 11-point Likert-type scale ranging from 0 (totally no trust) to 10 (totally trust).

The Chinese version of the Startle, Physiological arousal, Anger, and Numbness scale (SPAN) was employed to assess PTSD (Chen *et al*., 2003). This 4-item scale was rated on a 5-point Likert-type scale ranging from 0 (not painful or disturbance) to 4 (extreme painful or disturbance), and a total cut-off score of 5 and above was considered to indicate PTSD. Previous clinical research has demonstrated that SPAN is reportedly up to 88% correct compared to the 17-item Davidson Trauma Scale (DTS) and a clinical interview for assessing PTSD and is considered a better diagnostic screening tool (Meltzer-Brody *et al*., 1999). The scale in the current study presented an acceptable internal consistency (α = .642).

The scale to assess the challenges experienced during the pandemic was designed with 16 items rated on a 5-point Likert-type scale ranging from 1 (never) to 5 (always), and items covered challenges in areas related to daily life. The included items were based on challenges identified in previous literature or studies on pandemics (Brose *et al*., 2021; Chasiotis *et al*., 2021; Chen and Olsen, 2022; Chen *et al*., 2021; Fraser *et al*., 2020; Liu *et al*., 2020; Yan *et al*., 2022), with consideration given to the local context of older people in Mainland China, as understood by the research team members who had years of experience in conducting survey research in the aging population. For the challenge variable related to ‘disease management and information,’ higher total scores indicated that participants had taken positive actions to gain and manage the information or methods related to disease prevention. For the other three challenge variables, ‘supplies, service access and safety,’ ‘abuse and conflicts,’ and ‘anger and fear’, higher scores presented higher levels of challenges faced.

### Data analysis

Data analyses were conducted using IBM SPSS Statistics for Windows/Macintosh, version 26.0 (IBM Corp., Armonk, N.Y., USA). The raw data were raked-weighted based on census information on the gender–age–education distribution of the Shenzhen population aged 60 years and above.

To validate the challenge scale and avoid overfitting, the unweighted dataset was randomly split into a calibration set (*n* = 874) and validation set (*n* = 337) with the suggestion that 20%–30% of the data should be used for validation (Calaf *et al*., 2020; Gholamy *et al*., 2018). Exploratory factor analysis (EFA) was used to evaluate the factorial validity of the proposed scale using the calibration set (*n* = 874). The Kaiser–Meyer–Olkin (KMO) test and Bartlett’s test of sphericity were used. Adequate sample size is supported based on KMO estimates >.70, and Bartlett’s test is significant (*p* < .01) (Field, 2013). As the items and factors are interrelated, the principal axis factoring method for extraction and the Promax method for rotation were employed (Kaiser, 1960; Schmitt and Sass, 2011). Factor loadings greater than .3 are the threshold employed in this study for retaining items (Merenda, 1997).

Confirmatory factor analysis (CFA) was used to evaluate the construct validity of the scale using the validation set (*n* = 337). Diagonally weighted least squares (DWLS/WLSMV), less biased towards ordinal observed variables (Li, 2016), were adopted to estimate the parameters. The following criteria indicated good model fit: comparative fit index (CFI) >.95, Tucker–Lewis index (TLI) >.95, root mean square error of approximation (RMSEA) <.06, standardized root mean square residual (SRMR) < .06. and *χ* ^2^/df ≤ 3 (Bentler and Bonett, 1980; Brown, 2015; Hu and Bentler, 1999; Kline, 2015; Satorra and Bentler, 2001; Schreiber *et al*., 2006). The internal consistency of the proposed scale was assessed using Cronbach’s alpha and McDonald’s omega (Dunn *et al*., 2014).

Hierarchical logistic regression was performed to identify the significant predictors associated with dichotomous variables of PTSD, with a cut-off value of 5 in the SPAN. Demographic variables (age, sex, and education) and physical health were entered in the first and second blocks, respectively, to reduce confounding effects, and the factors related to challenges during COVID-19 were included in the third block. To help better interpret the results, the effect sizes (d) of the significant predictors in Model 3 were calculated (Chinn, 2000). The effect size is considered small if less than .2, moderate if between .2 and .5, and large if greater than .5 (Rhea, 2004).

To understand the effects of the moderating factors in this study, hierarchical multiple linear regression was used, which examined how the variables related to trust would moderate the relationship between challenges and SPAN scores measured as an ordinal variable. Demographic variables and physical health were entered as confounding variables in the first step. Challenge and moderator factor were included in the second step. Their interaction terms were entered in the third step, which enabled the presentation of the change in *R* ^2^. Low (1 SD below the mean), mid (mean), and high (1 SD above the mean) effects of the moderators were adopted to conduct a simple slope analysis. To handle multicollinearity, the independent variables and moderators were centralized.

## Results

### Participants characteristics

Participants’ demographic characteristics are presented in Table 1. Among the 1,211 participants, 41.5% (*n* = 503) of them were found to have PTSD with a score of 5 or above.

**Table 1.** Participant demographic characteristics

		*n* (%)/ Mean (SD)
Gender	Male	590 (48.7%)
	Female	621 (51.3%)
Age		67.56 (6.263)
Education	Middle school and below	804 (66.4%)
	High school and above	407 (33.6%)

### Validity of items measuring challenges

The factorial validity of the 16-item challenge scale was evaluated using EFA with a calibration set (*n* =874). The factor analysis results showed KMO values and Bartlett’s test of sphericity for the 16-item challenge scale of.837 (*χ* ^2^ = 4,252.392, *p* <.001) and revealed four dimensions emerging from the items measuring challenges shown in Table 2. These four extracted factors explained the 58.67% of the variance.

**Table 2.** Results of the factorial validity of the challenge scale

	λ (factor loading)			
Feel difficult to obtain virus protection supplies			.429	
Be discriminated, or targeted for exclusion, or treated badly because of the COVID-19			.431	
Encounter a lot of obstacles when you go out because of health and safety concerns			.796	
Experience special obstacles when you receive or need social and public services			.848	
Have more family conflicts with each other because they are more at home	.347			
Suffer more verbal abuse from your family at home	.860			
Suffer more physical abuse from family members at home	.863			
Suffer abuse from family members at home due to financial problems	.736			
View the news of COVID-19		.549		
Seek information on how to protect yourself from infections		.709		
Know about the virus and ways to protect yourself from infection		.623		
Adapt well to the changes that a viral outbreak brings to daily life		.444		
Share information/news about the COVID-19 with others		.483		
Spread positive energy and messages to others (e.g., encourage others to stay positive during COVID-19)		.527		
Feel fearful because of COVID-19				.711
Feel angry because of COVID-19				.799
Dimensions	Abuse and conflicts	Disease management and information	Supply and Services Access and Safety	Anger and Fear

Table 3 shows the CFA results (*x* ^2^/df = 1.38, RMSEA = .034, 95% CI: .018∼.047, CFI = .980, TLI = .976, SRMR = .076) of the 16-item challenge scale with the validation set (*n* = 337). The 4-factor structure fulfilled all the cut-off criteria for a good model fit, showing a satisfactory model fit. Cronbach’s alpha and McDonald’s omega results indicated good internal consistency for the items (α = .82–.83; ω = .88–.89).

**Table 3.** Confirmatory factor analysis of the 4-factor challenge scale

Model	x^2^	df	x^2^/df	RMSEA [90% CI]	CFI	TLI	SRMR
Challenge-4	135.296	98	1.38	.034 [.018∼.047]	.980	.976	.076

*Note*. RMSEA = root mean square error of approximation, CFI = comparative fit index, TLI = Tucker–Lewis index, SRMR = standardized root mean square residual.

### Factors associated with PTSD

Table 4 presents the results of the hierarchical logistic regression. When the demographic variables of sex, age, and education were entered, only sex and age were found to be significantly related to PTSD. Compared with male participants of different ages, female and older participants were more likely to have PTSD. When self-rated physical health was entered into the second model, it showed no effect on PTSD. In contrast, a significant effect of sex and age on PTSD remained the same as in the first model. When all the challenge variables were added as the third block of independent variables, the significant effects of sex, age, and educational level remained, while all four challenge variables were significantly related to PTSD. Having more challenges in ‘supplies, services access and safety’ (OR = 1.787, 95% CI: 1.426–2.240), ‘abuse and conflicts’ (OR = 1.761, 95% CI: 1.420–2.184), and ‘anger and fear’ (OR = 1.233, 95% CI: 1.059–1.435) were associated with having PTSD, while the odds of having PTSD decreased by 38.8% (OR = .612, 95% CI: .484–.773) for a one-unit increase in ‘disease management and information.’ According to the criteria of effect size (d), challenges in ‘supplies, services access and safety’ (d = .321), ‘abuse and conflicts’ (d = .313), and ‘disease management and information’ (d = .271) had a moderate effect, while challenge related to ‘anger and fear’ (d = .116) had a small effect.

**Table 4.** Results of the hierarchical logistic regression analysis on PTSD

	Model 1		Model 2		Model 3		
	B (SE)	Exp(B) 95% CI	B (SE)	Exp(B) 95% CI	B (SE)	Exp(B) 95% CI	d
Gender – Female^1^	.431** (.133)	1.539 (1.186–1.996)	.446*** (.133)	1.561 (1.202–2.028)	.376** (.140)	1.456 (1.107–1.914)	.208
Age	.100*** (.010)	1.105 (1.083–1.128)	.096*** (.011)	1.101 (1.078–1.124)	.073*** (.012)	1.075 (1.051–1.100)	.040
Education – High school and above^2^	.227 (.137)	1.255 (.959–1.642)	.229 (.138)	1.257 (.960–1.646)	.330* (.151)	1.390 (1.035–1.868)	.182
Physical health			−.194 (.107)	.824 (.668–1.016)	−.124 (.114)	.883 (.706–.104)	
Supplies, service access and safety					.581*** (.115)	1.787 (1.426–2.240)	.321
Abuse and conflicts					.566*** (.110)	1.761 (1.420–2.184)	.313
Disease management and information					−.491*** (.119)	.612 (.484–.773)	.271
Anger and fear					.209** (.077)	1.233 (1.059–1.435)	.116
Cox & Snell *R* ^2^	.080		.083		.176		
Nagelkerke *R* ^2^	.108		.111		.237		

*Note*. ^1^Reference: Male, ^2^Referece: Middle school & below; *p < .05, **p < .01, ***p < .001.

The pseudo *R* ^2^ of the final model were.176 (Cox & Snell *R* ^2^) and.237 (Nagelkerke *R* ^2^), explaining the approximately 17.6% to 23.7% variation in the dependent variable (PTSD).

## Moderation analysis of trust

Table 5 presents the significant moderating effects identified in this study. Details of the results of simple slope tests are presented in Table 6.

**Table 5.** The results of significant moderating effects with the multiple linear regression

	B (SE)	B (SE)	B (SE)	B (SE)
Gender – Female^1^	.320 (.126)*	.341 (.123)**	.371 (.125)**	.343 (.124)**
Age	.034 (.011)**	.045 (.011)***	.030 (.011)**	.032 (.011)**
Education – High school and above^2^	−.261 (.131)*	−.269 (.130)*	−.265 (.131)*	−.281 (.130)*
Physical health	−.371 (.100)***	−.424 (.099)***	−.399 (.100)***	−.391 (.100)***
Supplies, service access, and safety	.600 (.100)***	.541 (.099)***	.550 (.100)***	.533 (.099)***
Abuse and conflicts	.511 (.097)***	.509 (.096)***	**.544** (.099)***	.516 (.097)***
Disease managementand information	**−.193 (.108)**	−.223 (.103)*	−.309 (.104)***	−.331 (.104)**
Anger and fear	.385 (.070)***	**.434** (.069)***	.420 (.070)***	**.434** (.070)***
Trust in authority	**.014** **(.045)**	**−.046** **(.044)**	–	–
Trust in medical professionals	–	–	**.108** **(.065)**	**.149** (.065)*
Trust in authority*Disease management and information	.143 (.054)**	–	–	–
Trust in authority*Anger and fear	–	−.299 (.050)***	–	–
Trust in medical professionals* Abuse and conflicts	–	–	−.234 (.108)	–
Trust in medical professionals* Anger and fear	–	–	–	−.240 (.063)***
△*R* ^2^	.005	.023	.003	.009
△F	6.691	35.385	4.750	14.314

*Note.* ^1^Reference: Male, ^2^Referece: Middle school & below; △: change between block 1 and 2; The bolded values are the centralized variables; *p < .05, **p < .01, ***p < .001.

**Table 6.** Results of simple slope tests (*n* = 1211)

Moderator	Independent variable	Level of moderator	B	t	Sig
Trust in authority	Disease management and information	low	−.386	−3.441	.001
		Mid	−.193	−1.790	.074
		high	−.001	−.008	.994
	Anger and fear	low	.835	8.445	.000
		Mid	.434	6.296	.000
		high	.032	.345	.730
Trust in medical professionals	Abuse and conflict	low	.765	5.006	.000
		Mid	.544	5.525	.000
		high	.323	2.509	.012
	Anger and fear	low	.660	7.016	.000
		Mid	.434	6.239	.000
		high	.207	2.321	.020

The significant moderation effects of ‘trust in authority’ were found in the relationship between the PTSD scores and ‘disease management and information’ (△*R* ^2^ = .005, △F = 6.691, *p* = .008), ‘anger and fear’ (△*R* ^2^ = .023, △F = 35.385, *p* = .000). The simple slope analysis indicated that a higher level of ‘disease management and information,’ which meant fewer challenges in this domain, was significantly associated with a lower PTSD score measured by SPAN for those with a low level of ‘trust in authority.’ In comparison, such an association became insignificant for those with a relatively higher level of ‘trust in authority.’ A higher level of challenges related to ‘anger and fear’ was significantly associated with a higher PTSD score at the mid and low levels of ‘trust in authority.’ Compared to the slopes (B parameter), the lower the level of ‘trust in authority,’ the stronger the effect of ‘anger and fear’ on the PTSD score.

Significant moderation effects of trust in medical professionals were found in the relationship between the PTSD score and challenges related to ‘abuse and conflicts’ (△*R* ^2^ = .003, △F = 4.750, *p* = .030) and ‘anger and fear’ (△*R* ^2^ = .009, △F = 14.314, *p* = .000). A higher level of challenges in ‘abuse and conflicts’ was significantly associated with a higher level of PTSD at all levels of trust in medical professionals. This indicated that the higher the level of trust in medical professionals, the weaker the effect of ‘abuse and conflicts’ on PTSD. Positive and significant correlations between ‘anger and fear’ and the level of PTSD were found at all levels of trust in medical professionals, indicating that the higher the level of trust in medical professionals, the weaker the effect of challenges related to ‘anger and fear’ on the level of PTSD.

## Discussion

This study examined the association between the challenges brought about by COVID-19 and PTSD and tested the moderating effect of trust with a large sample size (*n* = 1211). All defined domains of challenges were significantly associated with PTSD, and both ‘trust in authority’ and ‘trust in medical professionals’ showed a moderating effect between PTSD and at least two factors of challenges.

Base on the existing literature on the prevalence of PTSD after pandemics (e.g., SARS, MERS-CoV, H1N1), healthcare workers and infected individuals were the highest-risk groups and showed higher rates of post-pandemic PTSD (Yuan *et al*., 2021). Although Vrach and Tomar (2020) suggested that older adults are vulnerable to PTSD, specific research on older adults is limited. In the current study, the percentage of older adults with the PTSD symptoms among all participants was 41.5%, which is comparable to the figures (ranging from 38.3% to 46.2%) in the Chinese sample of healthcare workers and patients (Gao *et al*., 2006; Hong *et al*., 2009; Wang *et al*., 2021) and apparently higher than previous reports on the prevalence (ranging from 9.2% to 30.9 %) of PTSD in vulnerable aging sub-population (e.g., older parents who lost their only child and older survivors of the earthquake) (Yin *et al*., 2020; Zhang *et al*., 2015). The present study found that the odds of being identified as having PTSD increased with age. This further confirms Vrach and Tomar’s (2020) view that older adults are at high risk of PTSD during a pandemic outbreak.

The results of the present study confirmed that challenges during the pandemic were significantly associated with PTSD. The current study found that PTSD was more prevalent in older adults with higher education, possibly due to the reasons described in previous studies, such as those who were highly educated were more aware of the risks of the pandemic, leading to more psychological complications (Bonichini and Tremolada, 2021; Rattay *et al*., 2021; Sun *et al*., 2021; Walter and McGregor, 2020).

Serious challenges in ‘supplies, services access, and safety’ were significant predictors of PTSD. This finding is consistent with previous studies showing that the negative impacts of the pandemic on daily life and social activities are related to deterioration in mental health (Brose *et al*., 2021; Meyer *et al*., 2020). Owing to the susceptibility of older adults to the virus, there has been notable age-based discrimination during the outbreak, which has led to the abuse of older adults (Fraser *et al*., 2020; Han and Mosqueda, 2020). A strong association between abuse and PTSD was found in a Korean study, and the strong effect of ‘abuse and conflicts’ on PTSD symptoms was also confirmed in the current study (Choi *et al*., 2018). Similarly, ‘anger and fear’ as predictors of PTSD have been supported by evidence from previous studies (Wang *et al*., 2020). However, a lower level of ‘disease management and information’ was a predictor of PTSD, unlike previously reported findings. Zarocostas (2020) suggested that excessive news about the outbreak may trigger crowd panic, leading to psychological stress. Nevertheless, in the long term, information related to the pandemic can also decrease public anxiety and lower the uncertainty level of the public (Liu *et al*., 2020). It has also been found that more relevant information often leads to an increased perception of control and improves coping ability (Chasiotis *et al*., 2021; Echlin and Rees, 2002). The results of this study also support the idea that a higher frequency of ‘disease management and information’ can reduce vulnerability to PTSD.

The higher the level of public trust, the less the impact of challenges related to ‘anger and fear’ and ‘abuse and conflicts’ on PTSD. Moreover, when trust in authority is sufficiently high, challenges related to ‘anger and fear’ can be statistically considered to not affect PTSD. These findings provide further evidence of the positive role of public trust in alleviating the psychological hazards of the pandemic in regions with high levels of political trust (van Tilburg *et al*., 2021).

However, when the level of ‘trust in authority’ was high, the benefits of high levels of ‘disease management and information’ disappeared. Trust in authority was found to be an essential factor in risk perception, with trust in authority or confidence in protective measures to reduce perceived risk (Wachinger *et al*., 2013). However, Huurne and Gutteling (2008) reported that the higher the risk perception, the more frequent the information-seeking behavior. When people with high trust in authority seek information frequently, there may be a contradiction in their risk perception. Such cognitive dissonance may occur when a person’s behaviors and beliefs are inconsistent, creating a more serious psychological burden that may hedge the benefits of strict disease management and high information levels (Harmon-Jones and Mills, 2019). Despite a few exceptions, nurturing a stronger sense of trust in authority and medical professionals would ease the psychological stress and concerns.

## Conclusion

The current study identified predictors of PTSD among older adults during the pandemic and tested the moderating effects of trust in authority and trust in medical professionals. The findings fill a gap in the research on PTSD among older adults during the pandemic and reveal the critical role of public trust in China. Different aspects of the challenges arising from the pandemic have been identified as affecting the psychological health of older adults. This finding could provide caregivers and practitioners with guidance on helping older adults maintain their mental well-being during a pandemic, especially when the effects would be long-lasting because of the FTTIS policy (Find, Test, Trace, Isolate, and Support). Based on the findings of the present study, more attention should be directed towards vulnerable subgroups, particularly women and those who are older, and targeted measures are needed to improve their mental health. Moreover, facing severe challenges related to ‘supplies, service access, and safety’ was found to be a significant predictor of PTSD; therefore, life support for the aging population is recommended to ensure that their daily lives are not affected during the pandemic. With the findings of having a lower level of challenge related to ‘disease management and information’ as a protective factor for PTSD, the timely release of accurate and effective information is recommended. As the results show that trust in authority and medical experts helped mitigate the adverse effects of the challenges experienced during the virus outbreak, it is important to make additional efforts to foster public trust among older adults. Previously, researchers (Gille *et al*., 2022) proposed guiding principles for the health system to enhance public health, highlighting that trust-building involves both emotional and rational thinking. According to the principles and findings of the present study, we would like to provide the following suggestions for policy makers and medical experts: 1) provide greater autonomy to the public to reduce the impact of preventive measures on daily lives and 2) strengthening three-way communication to improve mutual understanding among government, medical experts, and the public. First, in developmental psychology theory, greater autonomy is considered a sign of a healthy mindset and function, and ensuring public autonomy is conducive to maintaining psychological well-being (Bergamin *et al*., 2022; Ryan *et al*., 2016). In addition, a previous study reported that effective communication helps maintain public trust (Henderson *et al*., 2020).

## Limitation

Despite employing a sampling strategy that encompassed multiple locations in Shenzhen and using a large sample size to secure statistical power, it is important to acknowledge some limitations associated with this study. First, the current study did not comprehensively examine socioeconomic factors as significant predictors of psychological problems. Future research should include a wider range of participants with diverse demographic backgrounds (e.g., income, occupation). Second, despite previous research supporting the use of a single item as acceptable, valid, and repeatable (Yohannes *et al*., 2011), physical health was only assessed using a single-item and self-rated scale, which may have introduced some subjective biases. In future studies, multiple dimensions of health that may reveal specific needs, particularly for those with long-term and chronic illnesses, should be used, as they may be related to the various challenges that surface during the pandemic. Another limitation in the use of the scales that warrants attention is that the SPAN used to measure PTSD levels in this study contained only four items. Although the validity of the SPAN has been demonstrated, the use of clinical scales in future studies is more recommended. Third, as a cross-sectional study, the longitudinal impacts of the challenges and associations with specific policies and measures instituted during the pandemic could not be covered, and it is difficult to determine if the results have a temporal contingency.

## Data Availability

N/A.

## Data deposition

N/A.
